# Community Engagement in Long Covid: Insights From the Boston COVID Recovery Cohort

**DOI:** 10.1111/hex.70493

**Published:** 2025-12-02

**Authors:** Marisha E. Palm, Callie A. Gu, Ingrid V. Bassett, Bruce D. Levy, Li Qing Chen, Janice John, Cheralyn Johnson, Jacqui Lindsay, Rebecca Lobb, Netia McCray, Bisola Ojikutu, Linda Sprague Martinez, Jacqueline Rodriguez‐Louis, Alice Rushforth, Robert Torres, Danielle L. Zionts, Cheryl R. Clark

**Affiliations:** ^1^ Tufts Clinical and Translational Science Institute (CTSI) Tufts University Boston Massachusetts USA; ^2^ Institute for Clinical Research and Health Policy Studies Tufts Medical Center Boston Massachusetts USA; ^3^ Department of Medicine Brigham and Women's Hospital Boston Massachusetts USA; ^4^ Division of Infectious Diseases, Massachusetts General Hospital Boston Massachusetts USA; ^5^ Harvard Medical School Boston Massachusetts USA; ^6^ Cambridge Health Alliance Cambridge Massachusetts USA; ^7^ North Shore Community Health Salem Massachusetts USA; ^8^ Innovation by Design New York New York USA; ^9^ Boston University Clinical Translational Science Institute Community Engagement Program Boston Massachusetts USA; ^10^ Mbadkika Cambridge Massachusetts USA; ^11^ Division of Infectious Diseases Brigham and Women's Hospital Boston Massachusetts USA; ^12^ Division of Global Health Equity Brigham and Women's Hospital Boston Massachusetts USA; ^13^ The Health Disparities Institute UConn Health Hartford Connecticut USA; ^14^ Departments of Medicine and Public Health Sciences UConn Health Farmington Connecticut USA; ^15^ Division of Pulmonary and Critical Care Medicine Brigham and Women's Hospital Boston Massachusetts USA; ^16^ Beth Israel Lahey Health Cambridge Massachusetts USA; ^17^ Medical Practice Evaluation Center, Massachusetts General Hospital Boston Massachusetts USA

**Keywords:** community engagement, community leadership, community partnerships, community‐based organisations, long Covid, network building

## Abstract

**Background:**

In 2021, the National Institutes of Health launched a multi‐centre observational study on long Covid: Researching COVID to Enhance Recovery (RECOVER). Six Boston academic medical centres joined community partners to become the Boston COVID Recovery Cohort (BCRC), a consortium of RECOVER sites. Our consortium developed a community engagement model, and this manuscript shares lessons and recommendations.

**Design and Participants:**

The BCRC Community Partnership Table, which included community partners, senior equity leaders, academic researchers and health system collaborators, co‐developed a charter to advance research, community education, clinical care, social support and institutional and policy change goals. BCRC engaged patients, providers, caregivers and legislators via multiple communication channels.

**Findings:**

The BCRC Community Partnership Table faced several challenges: working within a novel, evolving pandemic; structural barriers to successful community engagement; perspectives on trustworthiness of research; and working across multiple organisations with distinct structures, resources and goals. There were also successes: leaders who were invested in community engagement; a focus on inclusive network building; co‐production; flexible communication channels; a shift to centring communities and patients; and connection with the legislature to support broader policy impacts.

**Discussion:**

To inform future community engagement models, we recommend the following: (1) healthcare research funders should build in time and resources for community engagement; (2) study consortia should include community engagement specialists in decision‐making positions from the outset; and (3) community members should have prominent roles leading research engagement efforts.

**Conclusions:**

Engagement models can enhance the equity impact of long Covid research. Reflections and recommendations in this paper can inform future efforts.

**Patient or Public Contribution:**

The project included community leaders, community‐based organisations, people with long Covid, and those caring for people with long Covid. Community leaders, community‐based organisations and people with long Covid are included in every aspect of the network. They inform decision‐making, play a key role in network leadership and are also all represented within the authorship team.

## 1 Introduction

The Covid‐19 pandemic began in December 2019, and by 2020, it became clear that, while most people recover after they are infected, others experience persisting post‐acute symptoms, now known as long Covid [1, 2]. In 2021, the National Institutes of Health published a Research Opportunity Announcement (ROA) for Researching COVID to Enhance Recovery (RECOVER), a national multi‐centre observational study to learn more about long Covid, why people get it, and how they recover [3]. The language in the ROA recognised the disproportionate impact Covid‐19 had on African American, Native American, Latine and other racial and ethnic groups [4] and proposed to involve communities and the affected patient population in the governance, design and execution of the research. To apply for this opportunity, six Boston academic medical centres—Beth Israel Deaconess Medical Center, Boston Medical Center, Brigham and Women's Hospital, Cambridge Health Alliance, Massachusetts General Hospital and Tufts Medicine—began discussions amongst themselves and with their community partners. These conversations included the local and state departments of public health, community health centres, faith‐based organisations and community‐based organisations. Boston RECOVER principal investigators engaged community partners in the submission planning process through meetings and email communications, and several partners provided letters of support that were included in the grant application. The proposal was successful and led to the enrollment of 893 participants from the greater Boston area. It also set the stage for the development of a community engagement model to support activities and host events designed to advance equity in RECOVER participation and to connect local communities to resources related to equity and long Covid.

This paper describes the development of the Boston COVID Recovery Cohort (BCRC) [5] community engagement model, including network building, co‐development of infrastructure, and supporting next steps in community leadership. Our authorship group includes individuals from each of the six Boston academic medical centres, as well as long Covid patients, policymakers, health equity researchers and representatives of community‐based organisations. As a collective, we believe it is important to be transparent about the challenges faced, as well as the successful strategies we identified, when developing our engagement model. In this paper, we share lessons learned and make recommendations for future design and implementation of community engagement initiatives. The lessons learned and recommendations shared are often generally applicable but may be most relevant for long Covid research.

## 2 Materials and Methods

### 2.1 Community Engagement Network Building

When the BCRC was launched, its three co‐equal goals were to: support the scientific aims of the RECOVER study; advance health equity; and centre the community's knowledge, voice and leadership in the research. To develop the BCRC community engagement model, we leveraged existing networks and built new partnerships to create an ecosystem of collaboration. As healthcare and health promotion entities within the city of Boston, each of the six health systems had active relationships with community groups, including neighbourhood health centres, health policy collaborators (e.g., Disability Policy Consortium), health equity research groups, foundations and faith‐based organisations. These community partnerships served as a strong starting point for further network building.

When Boston RECOVER was funded, an established Boston‐area community leader (J.L.) was recruited by study PIs to lead the community engagement work. The expertise that the community leader brought to this pivotal role included experience in community engagement with researchers and policymakers and content knowledge from prior support of a community‐wide coalition to increase awareness of Covid‐19. The prior role involved providing resources for Covid‐19 prevention, including education, community conversations, distribution of personal protective equipment (PPE) and vaccine access. The community leader first assembled a BCRC engagement group, with PIs designating equity and engagement leads from each health system to support the development of an engagement model. The group established a weekly meeting cadence throughout the course of the study to develop and implement an engagement strategy.

Post‐award study network building involved re‐engagement of existing community partners and a cross‐organisational effort to map greater Boston area communities to develop a list of diverse neighbourhood and community groups. Each of the five BCRC organisations had pre‐existing partnerships with several community groups. In addition, contacts within communities that had the highest incidence of Covid‐19 (e.g., elders, people of colour and those experiencing disability) were identified. Outreach was handled strategically, with some organisations and individuals contacted by the community leader and others contacted by those close to particular issues or organisations. Site PIs participated in these efforts, using their connections and positions to engage community groups where appropriate.

During the initial few months of grant funding, BCRC investigators, faculty and staff held interactive meetings to share information about the RECOVER study and to provide opportunities for community members and groups to help shape BCRC's work. On an ongoing basis, the key findings from interactive meetings were distilled and shared with community partners, senior equity leaders and health system collaborators working across the greater Boston area so they could provide feedback. As part of this iterative process, the group created a collaborative leadership structure named ‘The Community Partnership Table’ (hereafter referred to as the Table). The name included the term ‘partnership’ rather than ‘advisory’ to more accurately reflect the Table's participatory engagement in the study's mission. The Table was established as a way to support power sharing among BCRC interest holders. It is chaired by a community leader and works to drive collective impact by (1) identifying a shared vision, (2) ensuring that BCRC engagement activities support this vision and (3) involving interest holders in ongoing conversations about priorities and direction. The Table co‐developed a charter that outlines their mission, goals and related priority actions (see Table 1).

**Table 1:** Contents of the co‐developed Table charter.

The mission: Centring community and social justice to attain equity in long Covid care and recovery outcomes			
Goal		Description of goal	Actions taken by BCRC to reach the goal
1	Research	Diversify study recruitment, inclusive of those most severely impacted by long Covid	BCRC led community listening sessions and provided site PIs with recommendations for broader outreach for study recruitment that included diverse communities with the highest incidence of Covid‐19 infection. BCRC also facilitated engagement of community partners to aid with RECOVER outreach.
2	Community Education	Serve as a structure to support bidirectional communication and collaboration by centring accessible community education, relationship building and community building	BCRC convened Community Educational Forums (5 to date) on long Covid and health equity. Topics were informed by community‐level data and included: clinical care, resources and services; healing and recovery; and policy priorities to advance long Covid care and health equity. BCRC also sends quarterly newsletters and maintains an updated website to share national and local long Covid‐related news, events and resources.
3	Clinical Care and Social Support	Learn from Boston's diverse communities what long Covid clinical care and social support resources are available and what is needed to improve the healthcare and health of long Covid patients and their communities.	BCRC worked with long Covid clinics and patient and caregiver support groups to understand unmet needs, fostered collaboration between clinics, and connected health care organisations with community organisations to improve care equity.
4	Institutional and Policy Change	Build community relationships with hospital and policy leaders, learn more about and participate in advocating for institutional and policy change to more effectively address long Covid care, and advance health equity in our community, our sites, and our country.	In collaboration with long Covid patient advocates, long Covid equity researchers, BCRC community partners and long Covid clinic providers, BCRC identified 6 community‐driven policy priorities and held a closed‐door briefing with Massachusetts legislators, followed by a public briefing, to jointly advocate for equitable support for those living with long Covid.

The charter set the course for BCRC engagement work and served to hold partners accountable to the goals and actions established by the community. When it was initially established, the Table was nested within the BCRC research infrastructure, with BCRC RECOVER PIs and investigators directing the agenda. As the Table and its agenda matured, it took on additional autonomy, with community leaders driving an engagement and equity agenda that further advanced and complemented the research agenda. The Table meets quarterly to share progress towards goals outlined in the charter. The BCRC engagement group acts as a convener and facilitator for the Table meetings, bringing community groups together to share information, cross‐fertilise ideas and make connections.

**Figure 1:**
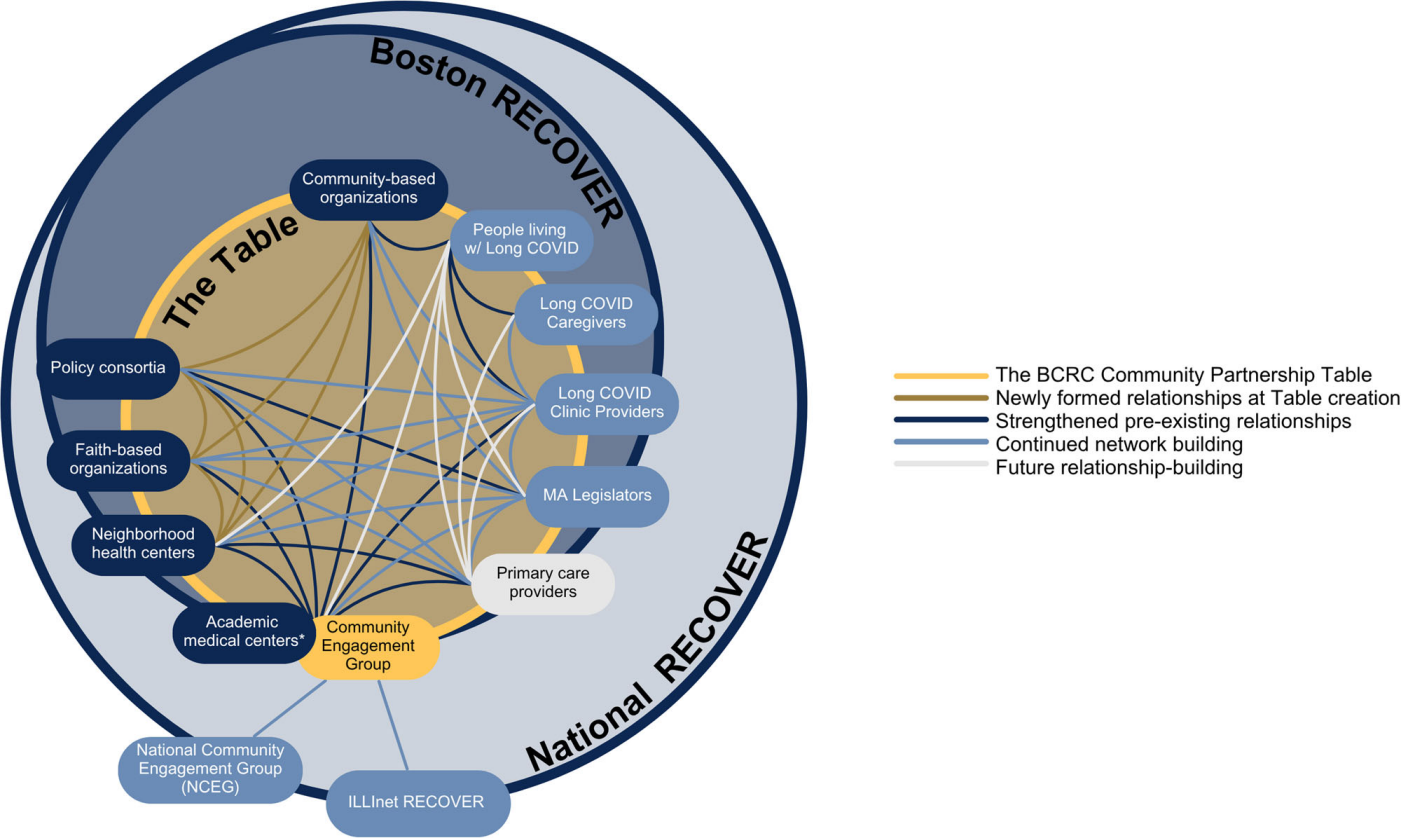
Boston COVID Recovery Cohort (BCRC) model and network building, including BCRC's positioning within national RECOVER, the stakeholder groups engaged with the BCRC Community Partnership table, and the relationships between stakeholders that were fostered and strengthened over time (*includes researchers, community engagement leads and senior leadership).

Across all the groups involved in the BCRC, existing relationships have been strengthened, new relationships have been developed, and there are ongoing efforts to continue building relationships (see Figure 1). Pre‐existing relationships were partnerships that existed between BCRC institutions and community groups before RECOVER's launch. New relationships were developed as a result of early community mapping efforts and strategic engagement related to the research study. Continued network‐building efforts fill gaps identified in current partnerships as part of an ongoing search for opportunities to grow in ways that meet the Table's mission and goals.

While BCRC worked at the local level in Boston to build an engagement network, national RECOVER leadership established the National Community Engagement Group (NCEG) at the consortium level. NCEG is a diverse group of 26 RECOVER community representatives, long Covid patients and long Covid caregivers from across the country who meet monthly. Initially, the work of NCEG was directed by national leadership, and meetings were used to keep members aware of RECOVER's progress in meeting its research goals. As the study evolved, NCEG sought to ensure that RECOVER research meets the needs of patients, caregivers and community members by sharing patient and community points of view, providing feedback on study materials and information collection, identifying ways to share study findings, and supporting study leaders to follow guiding principles around engagement.

First as a participant and then as a co‐chair, BCRC has been actively engaged in the work of NCEG. BCRC's efforts intersect with the work of NCEG by creating local networks of diverse community groups and other interest holders. Local Boston efforts support patient‐centred practices and work towards more equitable healthcare in the Greater Boston area and beyond.

### 2.2 Network Communications and Activities

**Figure 2:**
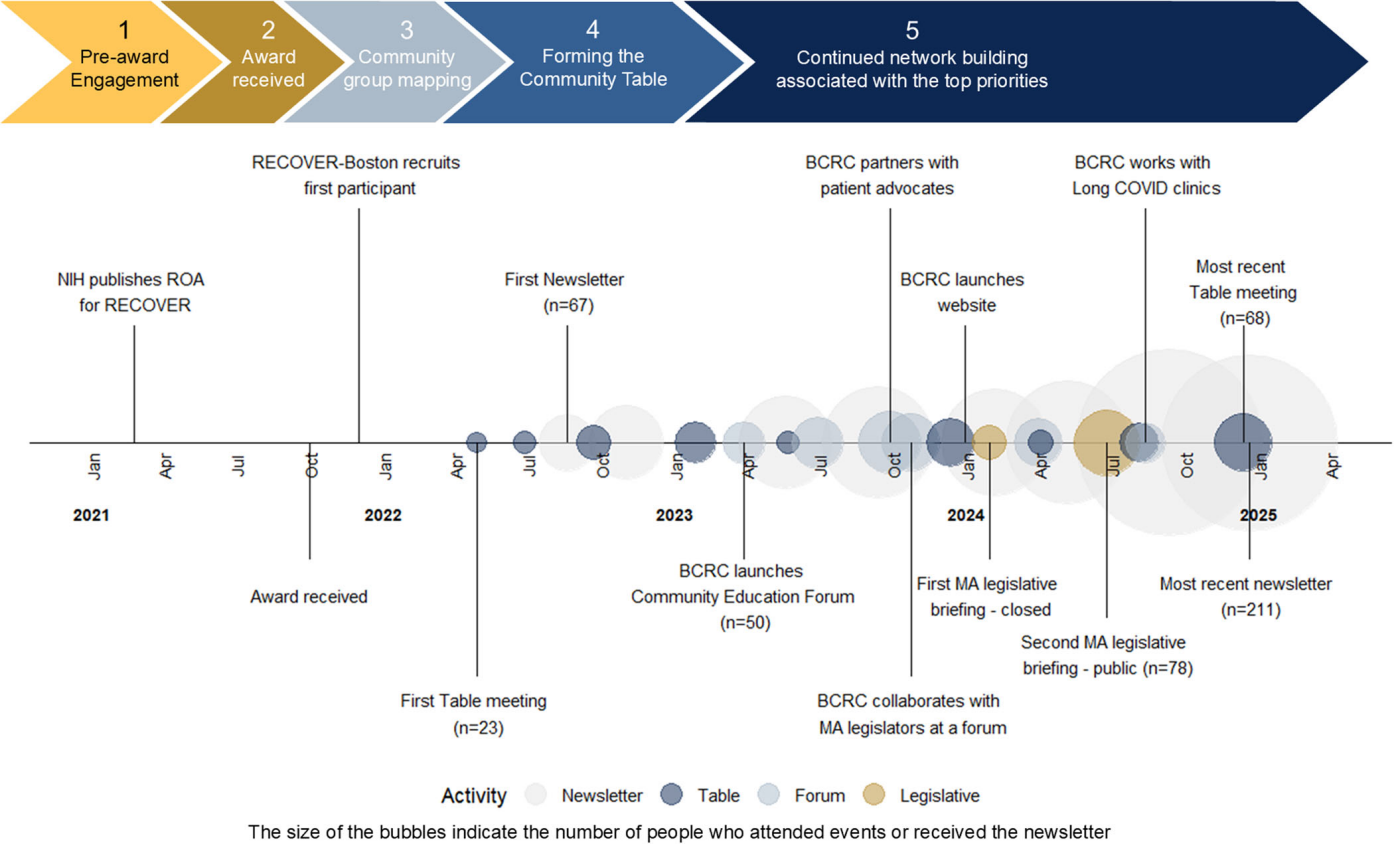
Community engagement timeline illustrating the growth of BCRC's outreach and the variety of engagement activities coordinated over time. Major milestones are indicated by text markers. The colours represent the type of activity, and the size of the bubbles indicates the number of people who attended the event or received the newsletter.

The partners who collaborated to build the community engagement model felt it was important to develop transparent communication pathways to connect members of the Table to each other and to the wider Boston‐area community. Communication venues were developed to inform the community about long Covid and the RECOVER study process, engage community partners, and share opportunities to get involved. This study was informed by data collected by the Massachusetts Consortium on Pathogen Readiness (MassCPR) Long COVID Equity group [6], which included RECOVER community engagement researchers among others. A BCRC newsletter, Educational Forum series, web page and legislative briefings were developed as complementary ways to support the engagement of diverse partners, including community groups, researchers, clinicians, those with long Covid, and policymakers (see Figure 2 for the timeline of events and activities).

In December 2022, the BCRC newsletter was launched and circulated to the Table membership. It included information about the RECOVER study documentation, long Covid clinical and community resources, a synthesis of the community feedback received, membership information, dates of the Table meetings, and community engagement work in progress. Since its launch, the newsletter has developed from an initial circulation of a Word document to an e‐newsletter service that provides the community with centralised quarterly updates. As of the fourth quarter of 2024, newsletter reach has increased by almost 300% from its launch to over 200 people, and a community spotlight has been added to showcase work being done by community partners.

The Community Education and Engagement Forum Series was introduced two months after the newsletter, in February 2023. The Forum Series is focused on topics that are aligned with the Table's mission and was developed to support the goals and priorities set by the Table. It functions to engage community partners, share relevant information, continue to build community networks, and support action to reach health service and equity goals. To date, attendance, which began at 23, has increased by 290% to approximately 64 people per forum, with 73 people in attendance at its height. Attendees include patients and members of the public, faculty and staff from Boston academic medical centres and smaller hospitals, individuals from community‐based organisations, and representatives from the Massachusetts Department of Public Health. The diversity of speakers and panelists has grown, and feedback gathered at the Forum Series has been used to shape future events as well as the direction of engagement efforts. For example, requests from Table members to discuss healing and recovery as a community resulted in a session to explore the power of community in transforming grief into resilience, anti‐fragility and advocacy [7, 8].

With a growing network, there was an increased need for an online platform to direct interested partners, share updates and host videos of events. In December 2023, the BCRC launched a new public‐facing page on the Boston RECOVER website to share information, showcase the Forum Series and build a place for future dialogue [5].

The most recent addition to our communication and collaboration efforts is a series of legislative briefings that began in January 2024. Leveraging relationships developed by the MassCPR long COVID Equity Core early on, the BCRC engagement group collaborated with Massachusetts legislative partners and the Table partners to develop a legislative briefing to educate policymakers about long Covid and issues related to health equity. This allowed continued co‐creation of a broader agenda that meets the Table's goals and priorities and aims to build a movement that will support and sustain health equity in long Covid care beyond the period of the NIH‐funded RECOVER study.

## 3 Results

### 3.1 Learning From Challenges and Successes

The BCRC community engagement model was co‐developed in a mission‐driven way, with broad representation from interest holders. It is structured to build shared understanding, engagement, trust and relationships among partners across sectors and communities. Our manuscript writing group includes the diversity of perspectives reflected in the Table: principal investigators, clinicians, researchers, engagement experts, policymakers and long Covid patients. As a group, we met three times to: synthesise the development of the BCRC engagement model; explore its challenges and successes; and discuss recommendations for funders, researchers and communities.

### 3.2 Community Engagement Challenges

We considered contextual, structural and cultural aspects of building the BCRC engagement model and identified four significant challenges. The fact that we were working within a novel and evolving pandemic was one of the most significant contextual challenges. This was true not only for the BCRC community engagement work but also for the research and data collection efforts. When the work began, Covid was a largely unknown entity; there was no clinical agreement on the definition of long Covid, and there was ongoing evolution in healthcare provider knowledge. Initially, the eligibility criteria would not allow inclusion of people who were reinfected, which meant that people in at‐risk communities were often ineligible. RECOVER study and care protocols evolved, and the enrollment process became more inclusive as our knowledge advanced; however, the rapidity of change and urgency felt by funders, clinicians, study investigators and communities made it challenging to align the needs for rapid study enrollment with the deliberate process of community building and the definition and pursuit of community‐driven goals.

After the first year, Table attendance evolved as the Covid health crisis became less about preventing the spread of infections and more about understanding the post‐acute sequelae that prevented people from returning to work or resuming daily living activities after infection [9]. With the diminished urgency of a pandemic, there emerged a post‐pandemic urgency of long Covid. Attendance among public health and policy leaders increased as the public health imperative of long Covid became more apparent. In addition, the emergency funds for Covid were depleted, and no funding for long Covid had yet been allocated to public health budgets.

The second major challenge to the development of an engagement model was structural barriers, including a lack of time, resources and funding to support successful community engagement. The initial funding call emphasised social justice and equity work and identified a role for communities in the operations of RECOVER; however, community and equity efforts were not always explicit areas of focus in funded RECOVER study processes. For example, there was a lag time in linguistic translation of study materials, which contributed to delays in recruitment of a more diverse group of participants. Structural challenges meant that expectations had to be reduced in scale and the scope of goals aligned with what could be delivered with the infrastructure and resources available. The reduced ability to address advocacy from Table members to improve study inclusion, and to enhance resources to pursue community goals for education, clinical care and institutional and policy change, was an initial impediment to strengthening community involvement in engagement activities.

A third major challenge was the perceived trustworthiness of research within some of the community groups related to past harms, power dynamics and infrastructure deficiencies [10]. Incomplete alignment of study activities and community engagement processes may have exacerbated community partners' perceptions of lacking a meaningful role in the project, amplifying power dynamics and reinforcing a perception of infrastructure deficiencies. While community partners were initially interested in supporting RECOVER, challenges in sustaining meaningful engagement likely contributed to a narrowing of community‐based support for the research effort.

A fourth major challenge was that the BCRC was working across multiple organisations with distinct operational structures and resources and different goals. Boston has a high density of healthcare resources with an overlap in patient populations, and while collaboration across six independent systems was achieved, study resources did not completely support community involvement across all sites. As a result, connections with original institutional partners were incompletely maintained.

### 3.3 Community Engagement Successes

We identified six successes that supported the development of our engagement model. First, strong leadership was an essential element. The BCRC had senior co‐PIs who were invested in community engagement as a critical part of the RECOVER work. Additionally, BCRC PIs identified a Table leader with strong facilitation skills and ties to the greater Boston area community. Study co‐PIs also involved engagement experts across all Boston RECOVER study sites, which required them to reach beyond traditional research partners to recognise those with specific skill sets that included collaboration and boundary spanning. Having time investment and leadership support for community engagement from the outset of the work positioned the effort to grow from a solid foundation.

The second success was a focus on diverse and inclusive network building. The BCRC engagement group, headed by the community leader who was a trusted source of information on Covid, prioritised leveraging existing networks and then identifying and establishing new partners. A key aspect of this success was intentionally breaking down silos between organisations and disparate communities. There were strategic efforts by our community engagement leader, as well as BCRC PIs, to have productive conversations with community, academic and healthcare leadership and to build support for continued network growth. The Table partners were asked to identify potential interest holders who were not represented at early meetings. This allowed for growth in new directions and led to intentional inclusivity. The inclusive approach to network building not only broke down traditional silos but also changed the group's expectations for the priorities and potential outcomes of research.

A third success was the use of co‐production (collaboration that includes interest holders, e.g., patients, clinicians and policymakers to develop, design and implement the end product [11]) to build the engagement model. This began with the innovative collaboration among six Greater Boston health systems. When community partners were asked to join the RECOVER effort, their organisational efforts and personal assets contributed to shaping Table activities. As an example, specific co‐production activities included expanding education about long Covid and RECOVER to a faith‐based setting and adding community education sessions to invite clinicians caring for long Covid patients to describe their resources and approaches in community settings. The BCRC charter, which set out a clear mission and related goals, was developed by the Table of community partners, senior health equity leaders and health system collaborators. While initially partners joined the Table on the strength of relationships, alignment of interests and goals was essential to continue involvement in the longer term. There was an emphasis on the transparency of co‐production and the importance of subsequent accountability to the co‐produced goals.

Our fourth success was the development of communication channels that allowed for flexible engagement. We developed multiple pathways and venues for communication that served to connect community groups and other partners to the work being done by health system collaborators, community members, equity partners and policymakers. The Community Education Forum Series is a platform for engagement that was developed to provide information on long Covid efforts in a way that centres community voices. We used an agile approach to engagement that allowed partners to be involved along a continuum of engagement, from passive receipt of information to actively doing work that contributes to BCRC's mission and priorities. Remote meetings worked well to support engagement by recognising limitations of those dealing with potential fatigue related to long Covid and general anxiety around continued disease spread. In addition to the Table meetings and the Forum Series, the BCRC newsletter kept people informed and engaged and contributed to meeting attendance. For those who could not attend the meetings, the website was designed as a searchable hub where people could access videos and meeting summaries.

A fifth success was the intentional shift from a focus on academic medical institutions to centring on leaders from communities most impacted by long Covid and groups of people with lived experience of long Covid. The first community engagement priority in the RECOVER grant was to diversify the study pool to reflect the population of greater Boston. Despite the challenge of a rapid recruitment deadline, there were some early wins related to outreach to elders. Since that time, we have intentionally grown community leadership. The BCRC engagement group facilitated conversations at the Table meetings, Educational Forums and Policy briefings and then circulated surveys after events and in newsletters that supported co‐development and ongoing feedback. Purposeful network growth and cultivated relationships made this move to community leadership more inclusive and impactful. As the community has taken on a leadership role, the focus shifted with study progress from recruitment to education and policy action, and the Table meetings are less focused on RECOVER updates and increasingly centre updates from community voices.

A sixth success is the legislative briefings led by the BCRC engagement group. These briefings were spearheaded by the community engagement lead and facilitated by co‐PI relationships and the high‐profile nature of Covid, long Covid and the NIH‐funded RECOVER study. Legislators were aware of RECOVER and interested in evidence‐based updates on the disease epidemiology, societal impact and patient experience, as well as learning about community needs related to long Covid. Two legislative briefings were held to discuss evolving definitions of long Covid and to describe the utility of quantitative data infrastructure, qualitative data collection and investment in clinical care and social support. The briefings also included consideration of long Covid referral systems, education for public health and primary care providers, and investment in community‐building infrastructure. Two current bills related to long Covid and informed in part by the experience of the BCRC are being sponsored in the 2025–2026 Massachusetts legislative session: an act to improve access to healthcare for people with long Covid with a patient navigation pilot programme (HD3839) [12] and an act measuring the impact of long Covid in the Commonwealth by initiating long Covid data collection and engaging in active surveillance using the National Academies of Sciences, Engineering, and Medicine definition (HD3840) [13].

### 3.4 Impact of Engagement on RECOVER in Boston

The community engagement efforts of BCRC supported RECOVER in three specific ways. The first was championing outreach to local elder, disability and faith‐based groups to increase the visibility of RECOVER within these communities. The second was petitioning for study materials to support diverse communities, which included translation of study materials and fielding surveys that captured social determinants of health. The third was encouraging the study team to think about outputs of research that go beyond traditional research products. Broadening the scope of research products changed the paradigm from the standard focus on peer‐reviewed manuscripts to outputs that included policy change and community education. As a result of BCRC engagement, we had appropriately translated materials, achieved diversity in our sample and shaped some of the scientific outputs to consider social determinants of health and increase real‐world impact.

### 3.5 Recommendations

In summary, BCRC developed a community engagement model that is inclusive and transformative in its support for policy and systems change. To inform future efforts, we reflected on our challenges and successes, and we suggest the following recommendations for others pursuing community‐informed research.

**Health research funders** (federal and non‐federal) should build in both the time and resources, including funding, for active community engagement and collaboration in all research projects. This will require establishing structures that allow for direct engagement and relationship building. The importance of recruiting diverse populations into clinical research studies is well established [14, 15]; however, the expectation for increased diversity has not been fully supported by changes in research structure and funding. Provision of funding based on meeting the total number of recruitment goals risks incentivising quantity over quality in recruitment efforts of representative populations. We recommend: (1) requiring community engagement as an essential and scored aspect of funding applications that rely on community participation; (2) funding that supports early partnership building to allow direct engagement with the community and frontline workers in preparation for recruitment; (3) financing that incentivises the diversity that is pivotal to study success (e.g., adjusting for cost rather than flat per patient rates and funding for study material translation); and (4) tailored instructions for recruiting participants that reflect the demographics of the communities within which different study sites are located, as national diversity targets do not always align with demographics in specific sites.

**Study consortia** (e.g., large coordinating centres) should include community and engagement specialists in decision‐making positions from the outset. There can be a disconnect between the national consortia and the local satellite study sites, and consortia leadership can inadvertently restrict site function to reporting rather than shared decision‐making. Particularly when there is an effort to involve the communities affected by poor health, we recommend a collaborative working community leadership model. This should involve transparency in communication and leadership in direction so that the community voices are reflected not only in responding to decisions made centrally but also in informing decision‐making.

**Engagement leads** with pre‐existing strong connections to the represented communities have a strong advantage in facilitating the development of a diverse network of interested participants. Our efforts were significantly helped by involving a Table lead who was familiar with our local communities and able to assemble non‐traditional partners such as healthcare clinicians, investigators, patients, community organisation leaders and policymakers.

Commitment to equity in research requires that represented communities be involved in study leadership and that recruitment efforts support inclusion of the diverse members of the impacted communities. This commitment should include funding, time, priority, leadership opportunity and inclusive decision‐making.

## 4 Conclusion

The BCRC developed an engagement model with an ethos and structure that enabled collaboration to support essential community participatory research focused on a new and disruptive health condition (i.e., long Covid). We co‐developed aims, built infrastructure and communication pathways, and centred community voices, including those in marginalised communities. We built local, statewide and national partnerships and will continue to build our networks, deepen partnerships, develop collaborative structures and strategically identify additional funding. After four years, we are just beginning to see certain benefits from our community engagement work, such as connecting long Covid patients and caregivers with local legislators. As a convener, we have supported the development of a health equity work group on long Covid and brought together an advisory board of people with long Covid who will work with researchers, clinicians and policymakers. We will also continue to engage with NCEG, supporting the ongoing work to include community members nationally in RECOVER.

The development of community engagement models that support more equitable and impactful health research is essential to improving individual and population health. The reflections and recommendations here are shared to help inform future engagement efforts.

## Author Contributions

**Marisha E. Palm:** conceptualisation, investigation, writing – original draft, methodology, visualisation, writing – review and editing. **Callie A. Gu:** conceptualisation, methodology, visualisation, writing – review and editing, investigation. **Ingrid V. Bassett:** conceptualisation, investigation, funding acquisition, methodology, writing – review and editing. **Bruce D. Levy:** conceptualisation, investigation, funding acquisition, methodology, writing – review and editing. **Jacqui Lindsay:** conceptualisation, investigation, methodology, writing – review and editing. **Jacqueline Rodriguez‐Louis:** conceptualisation, investigation, methodology, writing – review and editing. **Li Qing Chen:** conceptualisation, investigation, writing – review and editing, methodology. **Janice John:** conceptualisation, writing – review and editing, methodology. **Cheralyn Johnson:** conceptualisation, methodology, writing – review and editing. **Rebecca Lobb:** conceptualisation, methodology, writing – review and editing. **Netia McCray:** conceptualisation, methodology, writing – review and editing. **Bisola Ojikutu:** conceptualisation, methodology, writing – review and editing. **Linda Sprague Martinez:** conceptualisation, methodology, writing – review and editing. **Alice Rushforth:** conceptualisation, methodology, writing – review and editing. **Robert Torres:** conceptualisation, methodology, writing – review and editing. **Danielle L. Zionts:** conceptualisation, investigation, methodology, writing – review and editing. **Cheryl Clark:** conceptualisation, investigation, funding acquisition, methodology, writing – review and editing.

## Ethics Statement

The research was a collaborative process that did not include the collection of data beyond the numbers of individuals, interest holder groups and organisations involved in the building of a community engagement network. Therefore, the research did not involve human subjects and did not require formal ethical approval.

## Consent

The authors have nothing to report.

## Conflicts of Interest

The authors declare no conflicts of interest.

## Data Availability

The authors confirm that the data supporting the findings of this study are available within the article. Additional information is available from the corresponding author (M.E.P.) upon reasonable request.
